# Modulation of the thermosensory system by oxytocin

**DOI:** 10.3389/fnmol.2022.1075305

**Published:** 2023-01-09

**Authors:** Ugo Zayan, Laura Caccialupi Da Prato, Françoise Muscatelli, Valéry Matarazzo

**Affiliations:** Aix Marseille University, INSERM, INMED, Marseille, France

**Keywords:** autism spectrum disorder (ASD), oxytocin, thermo-sensory response, neurodevelopment, atypical sensory response

## Abstract

Oxytocin (OT) is a neurohormone involved early in neurodevelopment and is implicated in multiple functions, including sensory modulation. Evidence of such modulation has been observed for different sensory modalities in both healthy and pathological conditions. This review summarizes the pleiotropic modulation that OT can exercise on an often overlooked sensory system: thermosensation. This system allows us to sense temperature variations and compensate for the variation to maintain a stable core body temperature. Oxytocin modulates autonomic and behavioral mechanisms underlying thermoregulation at both central and peripheral levels. Hyposensitivity or hypersensitivity for different sensory modalities, including thermosensitivity, is a common feature in autism spectrum disorder (ASD), recapitulated in several ASD mouse models. These sensory dysregulations occur early in post-natal development and are correlated with dysregulation of the oxytocinergic system. In this study, we discussed the potential link between thermosensory atypia and the dysregulation of the oxytocinergic system in ASD.

## 1. Introduction

Oxytocin (OT) is a nonapeptide mainly secreted by neurons located in both the parvocellular division and the magnocellular division of the paraventricular nucleus (PVN), as well as in the magnocellular division of the supraoptic nucleus (SON) and the accessory nuclei of the hypothalamus in mammals (Lawson et al., 2020). It is involved in many functions such as induction of parturition or lactation, social or reproductive behavior tuning, and modulation of energy balance (Yuan et al., 2020). It acts not only as a neurohypophysial hormone through secretion into circulation to conduct its peripheral effects but also as a neuromodulator in the central nervous system (CNS) through either synaptic or dendritic release as well as volume transmission release for which OT diffuses in the extracellular space to act on structures expressing its receptor (OTR) (Grinevich and Ludwig, 2021).

OT/OTR signaling emerges very early in the course of mammalian development and it is associated with a dynamic spatiotemporal OTR expression (Mitre et al., 2018). In rodents, OTR is detected in the brain during embryonic neurogenesis and peaks in the second week of life. Analyses of the human brain revealed that OTR expression begins to accelerate right before birth, with a peak level of expression occurring during early childhood (Rokicki et al., 2022). Moreover, in mice, OT-expressing neurons are settled at embryonic day E14.5 (Madrigal and Jurado, 2021). The immature form of OT is detected as early as E16.5 and the mature form is after birth (Sannino et al., 2017).

OT/OTR signaling dysregulation can have a drastic effect on neurodevelopment, suggesting that the oxytocinergic system during this critical window could be implicated in some neurodevelopmental disorders such as autism spectrum disorder (ASD) (Sannino et al., 2017). Such dysregulation of the oxytocinergic system has been demonstrated in different genetic models of ASD, and a recent meta-analysis of 31 studies suggests that children (but not adults) with ASD have lower blood oxytocin levels compared to neurotypical individuals (John and Jaeggi, 2021).

During early life, sensory systems are very important for the construction of future socio-cognitive and emotional behaviors (Grinevich and Stoop, 2018). The effects of OT on the development, maturation, and regulation of the sensory systems (Grinevich and Stoop, 2018; Wang et al., 2022) are mediated by experience-dependent plastic changes which are known to occur during the early childhood-adolescence window (Onaka and Takayanagi, 2021).

The implication of the oxytocinergic system to set up sensory functionalities during the early period and its downregulation in children with ASD could account for sensory processing atypicalities (i.e., hyposensitivity or hypersensitivity) (Robertson and Baron-Cohen, 2017). Atypia is experienced by 90% of patients with ASD and is experienced as early as a few months from the diagnosis, which makes it one of the earliest diagnostic criterion for this disorder (Grzadzinski et al., 2020). Moreover, knowing the impact of the sensory systems on socio-cognitive and emotional behaviors and the fact that patients with autism have an atypical sensory understanding of the surrounding environment could lead to altered socio-cognitive behaviors (Park et al., 2021). OT treatment might be beneficial for sensory processing (Hubble et al., 2017) and therefore could have a beneficial impact on some cognitive deficits (Kanat et al., 2017).

This modulation of sensory processing by the oxytocinergic system has been largely reviewed. In this study, we emphasize the pleiotropic effects of the oxytocinergic system on one overlooked specific sensory modality: thermosensory processing.

The thermosensory system allows us to sense external temperature variations and induce physiological and behavioral thermoregulation to maintain a stable core temperature. Sensing any drop in external temperature might become vital for mammalian newborns since, unlike their homeothermic adult counterparts, neonates are poikilothermic: their adaptation to external temperature is not yet fully established. This system also implies the ability to have the consciousness of the environmental temperature, namely temperature perception, which participates in our comprehension of the surrounding environment.

In addition, we summarize the different studies demonstrating the role of OT to modulate both physiological and behavioral thermoregulation. Atypical temperature perception as well as thermoregulation issues have been observed in ASD. Considering that both the oxytocinergic and the thermosensory systems have been found dysregulated in ASD, we hypothesize that OT dysregulation in ASD is a potential physiopathological mechanism of thermosensory dysfunction.

## 2. Part 1: OT effects on temperature sensing and regulation

**Figure 1:**
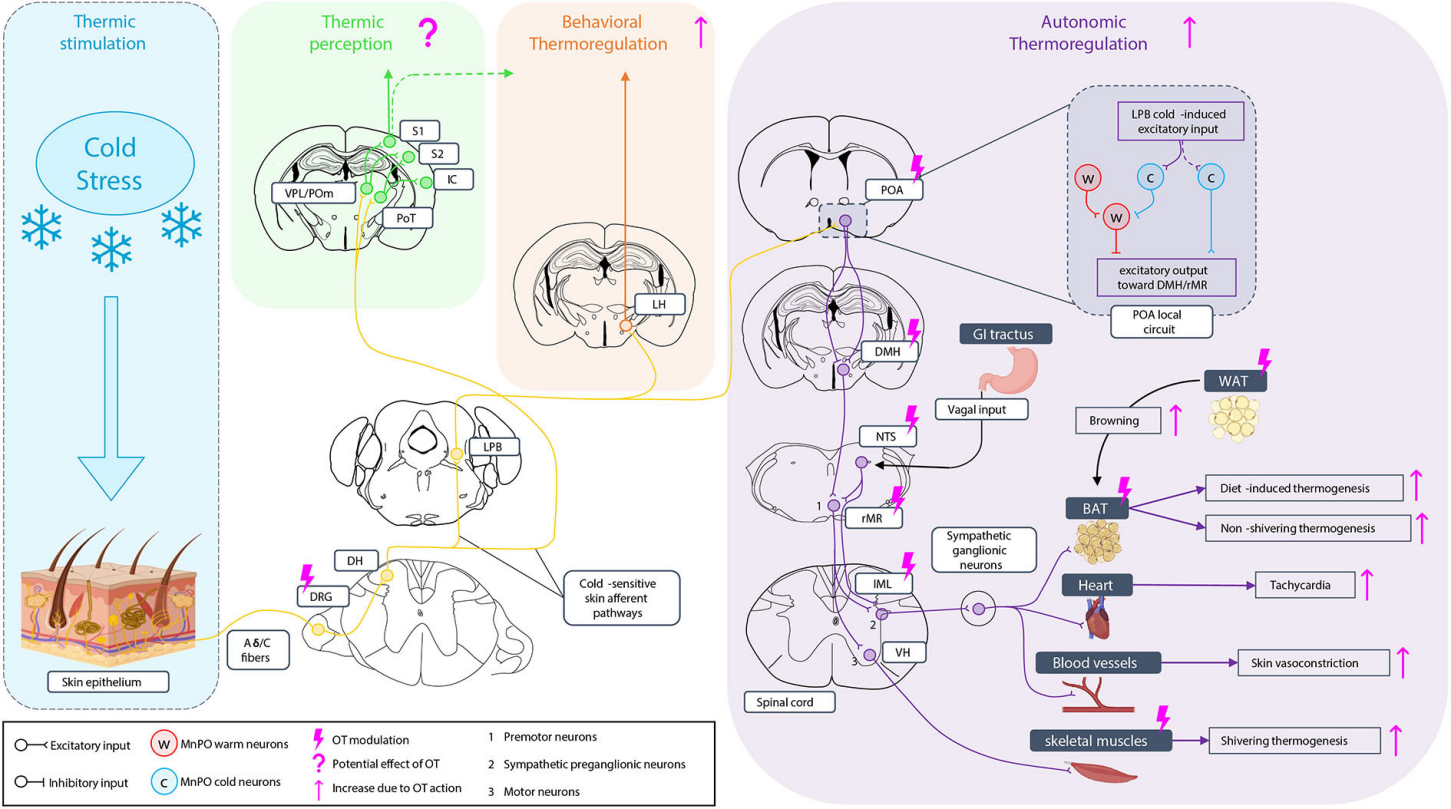
Oxytocinergic modulation of the thermosensory system under cold stimulation. Under cold exposure, skin thermoreceptors expressed at dorsal root ganglia (DRG) neurons ending are activated and the sensitive information is conducted to medullar neurons of the dorsal horn (DH). The information is sent through two types of cold-sensitive skin afferent pathways: i) in one case, to the posteromedial (POm), the ventral posterior lateral nucleus (VPL), and the posterior triangular nucleus (PoT) of the thalamus. The PoT project to the insular cortex (IC), the VPL, and the PoT project to the accessory somatosensory cortex (S2), and the VPL and the POm project to the primary somatosensory cortex (S1) giving the perception of temperature; ii) in the second case, the cold-sensitive skin afferents contact the lateral parabrachial nucleus (LPB) where the information is once more separated in two pathways. The first goes to the lateral hypothalamus (LH) to engage the behavioral thermoregulation. A potential implication of the somatosensory cortex in behavioral thermoregulation is not excluded. The other one is related to autonomic thermoregulation. LPB projection goes to the preoptic area (POA), an integrating center of temperature information with a local circuit in which the inputs from the LPB excite excitatory cold-sensitive neurons but also lead to the activation of cold-sensitive neurons of the median preoptic nucleus (MnPO) that inhibit warm-sensitive neurons, leading to an excitatory output of the POA toward the dorsomedial hypothalamus (DMH). Neurons of the DMH then project to the rostral medullary raphe region (rMR) which is also contacted by neurons of the nucleus tractus solitarius (NTS) receiving visceral vagal input, for the induction of the diet-induced thermogenesis. Premotoneurons of the rMR project then to sympathetic preganglionic neurons of the IML, activating the sympathetic ganglionic neurons to induce the non-shivering thermogenesis of the brown adipose tissue (BAT), the diet-induced thermogenesis on the BAT (related to NTS input), heart tachycardia, and vasoconstriction of skin blood vessels. Premotoneurons of the rMR project also to motoneurons of the ventral horn (VH) of the medulla to induce skeletal muscle shivering thermogenesis. Oxytocin (OT) can modulate behavioral thermoregulation without knowing yet whether it can act on LH. It can modulate the autonomic thermoregulation at different levels increasing BAT and muscle thermogenesis, tachycardia, and vasoconstriction but also by stimulating white adipose tissue (WAT) browning toward BAT to increase the thermogenesis capacity.

The thermosensory system is essential for the survival of individuals. It enables us to not only have a conscious perception of external temperature but also induce a physiological response of thermoregulation, leading to heat production and retention under cold exposure, namely thermogenesis, as well as appropriate behavioral strategies to maintain a stable body temperature. The neural pathways that support this ability have been intensively investigated in the last decade in adult rodents, but it has received much less attention in newborns. The thermosensitivity to external temperature starts at the skin level where different types of thermoreceptors are expressed (Patapoutian et al., 2003) and activated by different ranges of temperature (Dhaka et al., 2006; Palkar et al., 2015; Lamas et al., 2019; Buijs and McNaughton, 2020), allowing humans to differentiate between cool, warm, cold, and hot. The skin on the body and the face is innervated by first-order thermosensory neurons, located, respectively, in the dorsal root ganglia (DRG) or the trigeminal ganglia (TG) (Xiao and Xu, 2021) and projecting to the medulla. The sensory information is then transmitted through two different neural pathways leading to thermosensation and thermoregulation responses (Morrison and Nakamura, 2011). The thermosensation pathways which correspond to the conscious perception of external temperature variations are provided by the spinothalamic tract projecting to the somatosensory cortex (Milenkovic et al., 2014; Bokiniec et al., 2018) and also to the insular cortex (mainly for humans) (Craig, 2002; Filingeri, 2016). In parallel, another part of the projections from the medullary neurons reaches the lateral parabrachial nucleus (LPB) (Morrison et al., 2014) to coordinate the thermoregulation in response to environmental temperature variations (Nakamura and Morrison, 2011). Two types of thermoregulation responses are commonly defined. First, an autonomic involuntary thermoregulation response is mediated by descending projections from the preoptic area (POA), leading to activation through sympathetic pathways (Romanovsky et al., 2009) of different thermo-effectors. These effectors include the brown adipose tissue (BAT), the skeletal muscles, the heart, and the blood vessels that fight against temperature variations (Tan and Knight, 2018; Nakamura et al., 2022). Mammalian newborns are poikilotherms, and during this period only the BAT is recruitable for thermogenesis (Cannon and Nedergaard, 2004). The second thermoregulatory response implies a behavioral voluntary thermoregulation response using various behavioral strategies to allow mammals to stay as much as possible in a thermoneutral environment (Almeida et al., 2015; Jung et al., 2022). It has been shown recently that such behavior involves the lateral hypothalamus (LH) (Jung et al., 2022). These neural pathways are represented in Figure 1. Different studies investigating the action of OT on this thermosensory modality found that OT has a pleiotropic mode of action by tuning both central circuitry and peripheral thermos-effectors.

### 2.1. OT participates in maintaining stable core temperature through activation of non-shivering thermogenesis

Hypothalamic OT neurons are polysynaptically connected to the BAT (Oldfield et al., 2002), which produces heat in response to cold temperatures (Cannon et al., 1998). The descending projections arising from OT neurons of the paraventricular nucleus (OT PVN neurons) (Oldfield et al., 2002) innervate sympathetic preganglionic neurons of the intermediolateral (IML) nucleus of the spinal cord (De Luca et al., 1989; Bamshad et al., 1999; Foster et al., 2010), which express OTR (Reiter et al., 1994) and trigger BAT to produce heat through the sympathetic nervous system and β-adrenergic activation (Takayanagi et al., 2008). Besides this OT-regulated sympathetic action, endocrine regulation of the BAT by OT exists since BAT adipocytes express the OTR (Lawson, 2017) and it is known that OT can activate BAT's lipolysis pathway (Deblon et al., 2011). Additionally, different brain structures involved in thermoregulation, such as the POA (Shi and Bartness, 2000), the dorsomedial hypothalamic nucleus (DMH) (Shi and Bartness, 2000), and the ventromedial hypothalamic nucleus (VMH) (Whitman and Albers, 1998), express the OTR (Gimpl and Fahrenholz, 2001; Yoshida et al., 2009) and are innervated by OT PVN neurons.

Different approaches have been undertaken to study how OT modulates the capacity of the BAT to generate heat through lipid oxidation. First, pharmacological treatments with OT or analogous applied through different routes of administration have been performed to study their effects on BAT thermogenesis. In rats, OT injection in the anterior POA causes hyperthermia (Lin et al., 1983), and OT intracerebroventricular (ICV) administration in the third ventricle (3V) or fourth ventricle (4V) elevates interscapular BAT (IBAT) temperature (Roberts et al., 2017) and increases core body temperature (Ong et al., 2017). In mice, central OT injection produces a transient elevation of the colonic temperature (Mason et al., 1986), local application of OT into the median raphe nucleus (MnR) increases the body temperature (Yoshida et al., 2009), and ICV OT administration in mice 3V or 4V is reported to elevate IBAT temperature (Roberts et al., 2017). In rabbits, OT ICV injection produces dose-related hyperthermia and increases the body temperature (Lipton and Glyn, 1980). Roberts et al. (2017) demonstrated that the effect of OT on BAT thermogenesis is OTR-dependent since a lack of effect of the OT ICV injection in mice and rats was observed when the animals were pretreated with OTR antagonist. Thus, in different species, exogenous OT in the central nervous system has been shown to produce hyperthermia through a non-shivering thermogenesis response and without the elevation of locomotor activity for the rodent models (Deblon et al., 2011; Maejima et al., 2011; Blevins et al., 2016; Iwasa et al., 2019). Especially, peripheral OT administration in rats showed a reduced core body temperature (Ring et al., 2006; Hicks et al., 2014; Ong et al., 2017) as well as an absence of a thermogenesis response (Iwasa et al., 2019). Kohli et al. (2019) explained this contradiction by the fact that peripheral OT mediates its effects through the activation of arginine vasopressin (AVP) receptor V1A rather than OTR since pretreatment with V1A antagonist can reduce the OT-mediated hypothermia, while pretreatment with OTR antagonist does not.

Second, the development of OT knockout (KO) and OTR KO mice has been very useful for studying the impacts of a lack of OT/OTR signaling on thermoregulation. Hence, OT KO mice have difficulties maintaining their body temperature when they are exposed to cold temperatures (Kasahara et al., 2007; Harshaw et al., 2018). Moreover, exposure to cold results in c-Fos expression within OT PVN neurons, highlighting the implication of those neurons in thermogenesis induced by cold exposure (Kasahara et al., 2007). These results are in line with previous examinations of induced cold stress activation of the PVN (Baffi and Palkovits, 2000; Bratincsák and Palkovits, 2004). In the same line, the author demonstrated that OTR KO mice also failed to maintain their body temperature when exposed to cold (Kasahara et al., 2013; Harshaw et al., 2018). These results correspond with those of OTR KO mice harboring impaired thermoregulation (Takayanagi et al., 2008; Camerino, 2009). To directly link the deficit in thermogenesis with OTR signaling, the author rescued the expression of the OTR by injecting an AAV-Oxtr-IRES-Venus virus into the DMH and the VMH of OTR KO mice. This rescue compensated for some of the previous deficits in thermoregulation when the injected mice were exposed to a cold environment (Kasahara et al., 2013). Additionally, the authors proved the importance of the OT/OTR system in the rostral medullary raphe (rMR) for thermoregulatory function under cold exposure (Kasahara et al., 2015). The role of OT neurons in thermoregulation has also been studied in the diphtheria-induced ablation of these neurons. Upon cold exposure, OT-neuron–ablated mice showed a lower core body temperature, lower BAT response to cold, and a decrease in vasoconstriction in the skin, indicating once more the importance of the oxytocinergic system in thermoregulation (Xi et al., 2017). Pharmacogenetic stimulation of mice OT PVN neurons performed using DREADDs technology resulted in increased energy expenditure and IBAT temperature (Sutton et al., 2014). Similarly, a recent study in mice showed that the optogenetic activation of OT PVN neurons stimulates the sympathetic premotor neurons of the rMR through a direct connection, leading to an increase of the sympathetic outflow increasing both the BAT thermogenesis and the cardiac tachycardia (Fukushima et al., 2022). This increases the supply of oxygen and nutrients to the BAT, enhancing its thermogenic function and facilitating the systemic distribution of the heat generated by the BAT (Nakamura, 2011). To complement these data, a study of OTR gene expression after cold stress has been undertaken, showing upregulation of this gene in the brain, reinforcing the role of the oxytocinergic system in response to cold stress challenge (Camerino et al., 2018).

Thus, disruption of OT/OTR signaling results in thermogenesis deficits and the incapacity of mammals to maintain a stable temperature under cold environment exposure.

### 2.2. OT increases energy expenditure through BAT thermogenesis activation

Another approach to studying the effect of OT on BAT thermogenesis is to look at the effect of OT on energy expenditure, an indirect indicator of BAT thermogenesis activation. Energy homeostasis resides in the balance between energy intake that mainly comes from food consumption and energy expenditure which represents the basic metabolic processes and exercises. The process of BAT non-shivering thermogenesis is an important component of energy expenditure (Seale and Lazar, 2009; Saito, 2013). In addition to its thermoregulatory function in cold environments, the BAT can turn excess energy into heat to maintain the energy balance in rodents and humans (Nakamura and Nakamura, 2018). This process is called diet-induced thermogenesis (DIT) (Hibi et al., 2016). In the same manner, the assessment of OT's effects on energy expenditure has been addressed with the exogenous administration of OT. Oxytocin infusion in the third ventricle of mice (Zhang and Cai, 2011; Zhang et al., 2011) or OT injection in the VMH of rats (Noble et al., 2014) promote energy expenditure. Similarly, subcutaneous OT injection in DIO (Diet-induced obese) mice (Maejima et al., 2011) or chronic subcutaneous OT injection in DIO rhesus monkeys (Blevins et al., 2015) promotes energy expenditure. Moreover, the chemogenetic activation of OT PVN neurons that project to ChAT-positive neurons of the IML increases energy expenditure (Sutton et al., 2014). Conversely, mice harboring a viral-induced (synaptotagmin-4 overexpression) diminution of OT exocytosis (Zhang et al., 2011), or a diphtheria toxin-elicited reduction in OT PVN neurons (Wu et al., 2012; Xi et al., 2012), or mice treated with an OTR antagonist by ICV injection (Zhang and Cai, 2011), or mice harboring a deletion of OTR (Nishimori et al., 2008) or OT (Camerino, 2009) gene display a reduction in energy expenditure. The shRNA silencing of OTR expression in the nucleus tractus solitarius (NTS) has highlighted its role in diet-induced thermogenesis in a rodent model (Ong et al., 2017).

Collectively, these data indicate that OT/OTR signaling is a very important modulator of the thermogenic response to cold exposure by controlling BAT thermogenesis and energy expenditure. As Lawson et al. (2020) mentioned, the question remains what is the correlation between increasing energy expenditure and increasing BAT thermogenesis and the OT subpopulations that generate these activations.

### 2.3. OT browning induction of white adipose tissue

Another mode of action for OT to promote energy expenditure by increasing the thermogenesis capacity is through the “browning” of white adipose tissue (WAT). Nedergaard and Cannon (2014) define browning as the increase in the production of the uncoupling protein 1 (UCP1) in the WAT depots converting it to beige adipose tissue (Ishibashi and Seale, 2010), brite adipose tissue (Petrovic et al., 2010), convertible adipose tissue (Loncar, 1991), ectopic adipose tissue (Lehr et al., 2009), inducible adipose tissue (Lee et al., 2011), or recruitable adipose tissue (Schulz et al., 2013). This UCP1 protein is a marker of BAT associated with thermogenesis function and energy expenditure (Plante et al., 2015). This phenomenon of WAT browning was observed for the first time by Young et al. (1984), where the adipose tissue acquired the specificity of BAT when mice were exposed to cold.

Peripheral injection of OT in mice has been reported to increase the number of UCP1-expressing cells in visceral and subcutaneous WAT (Plante et al., 2015). Moreover, repeated cold exposure of mice has been shown to increase hypothalamic OT secretion with a significant elevation of plasma OT. This elevation of plasma OT increases both the expression of OTR and UCP1 in BAT as well as in inguinal WAT (IWAT) (Yuan et al., 2020). It is essential to remember that the effect of OT on BAT or WAT could also be mediated indirectly through the activation of the sympathetic nervous system (Blevins et al., 2015).

Furthermore, OT can differentiate adipocyte precursors into brown adipocytes after cold exposure (Yuan et al., 2020). In line with these results, OT infusion in mice's fourth ventricle elevates UCP1 expression in IWAT (Edwards et al., 2021). In athletic women, a high OT level has been reported to be associated with a secretion of hormones (irisin and FGF-21), leading to WAT browning and an increase in energy expenditure (Lawson et al., 2014). Conversely, Ott et al. (2013) showed that a single intranasal (IN) OT administration in men does not affect energy expenditure. Lawson et al. (2014) argue that the induction of browning and subsequently increased energy expenditure might require chronic high OT levels instead of transient OT elevation.

In addition to fat tissues, mice skeletal muscles mediating the shivering thermogenesis and expressing OTR (Gajdosechova et al., 2015) are also activated by peripheral OT, which is also able to upregulate the expression of genes related to heat production such as the uncoupling protein 3 (UCP3) (Yuan et al., 2020). Moreover, a cold exposure at 4°C as short as 6h has been shown to increase the expression of OTR in the soleus and tibialis anterior muscles (Conte et al., 2021), which potentiates the action of OT on these thermoeffectors.

Thus, Camerino (2021) mentioned that OT influences both shivering and non-shivering thermogenesis by regulating the expression of heat production-related genes in BAT, skeletal muscles, and WAT.

### 2.4. OT modulation of thermoregulatory behaviors

When exposed to cold, thermogenesis response is of two types: (i) involuntary autonomic thermogenesis (as discussed above) where OT acts directly or indirectly on the thermoeffectors, and (ii) voluntary behavioral thermoregulation whose function is to implement strategies to limit exposure to cold or heat loss in the case of a cold environment condition. It appears that this second aspect is also regulated by OT.

As previously noted, since locomotion is not yet fully functional until 10 days of age in rodents, autonomic thermogenesis is restricted to BAT non-shivering thermogenesis in neonates (Asakura, 2004). Therefore, this behavioral thermoregulation is an important component of their survival.

One of the behavioral strategies, called thermotaxis, which consists of animals moving from a cold or hot uncomfortable temperature toward a neutral temperature is lacking in OT KO mouse pups (Harshaw et al., 2018). Another thermoregulatory behavior called huddling, used by rodents' pups and adults (Alberts, 1978) to fight against the cold (Harshaw and Alberts, 2012), is also affected in OT KO mouse pups showing less cohesivity (Harshaw et al., 2018). These authors found that a deficit of BAT thermogenesis in OT KO pups contributes to the observed phenotype (Harshaw et al., 2018), a result confirming to that of Sokoloff and Blumberg (2001), showing that inhibition of BAT thermogenesis compromises huddling in rat pups. Moreover, as Harshaw et al. (2021) mentioned in their review, OT manipulation has been shown to impact huddling in different models of rodents such as meadow voles (Beery and Zucker, 2010), rats (Kojima and Alberts, 2011), naked mole-rats (Mooney et al., 2014), and mice (Arakawa et al., 2015; Tan et al., 2019). It is also true for marmoset in which IN OT administration increases huddling (Smith et al., 2010) and OTR antagonist treatment reduces it (Smith et al., 2010; Cavanaugh et al., 2018).

Under cold exposure, since pups do not yet have the capacity for thermotaxis, another strategy is the use of ultra-sonic vocalization (USV) to alert their warmth-giving dam (Portfors, 2007). We have previously showed that this thermoregulatory behavior is also modulated by OT (Da Prato et al., 2022). Indeed, we showed that IN administration of an OT agonist rescues the deficit of reactivity to vocalize under cold stress in an autistic mouse model presenting OT deficiency (Magel2^+/−p^).

Thus, another way that the OT can participate in maintaining a stable body temperature for the survival of the animal in addition to its modulation of autonomic thermoregulation is to enhance behaviors promoting heat retention. This is even more important for the poikilotherms newborns which are more vulnerable to cold exposure and subsequent lethal hypothermia.

### 2.5. OT effects on temperature perception

In comparison to adults, little information exists in newborns on the neural pathways supporting this thermal perception. Furthermore, from our knowledge, nothing has been published on the effect of OT on thermal perception except studies on pain induced by extreme temperatures. In the context of neuropathic pain caused by thermal hyperalgesia, OT has been shown to alleviate nociception by activating OTR-expressing GABAergic interneurons of the spinal cord (Sun et al., 2018). Furthermore, OT could have a direct effect on the DRG neurons to induce the analgesia of thermal pain. Such action is produced by activation of the vasopressin-1a receptor (V1a) rather than OTR, even if OTR is expressed in the DRG (Han et al., 2018). However, while OTR is known to be expressed in the somatosensory cortex (Son et al., 2022), the action of OT with thermoception in this cortical region has never been explored so far.

Thus, by signaling either through OTR or VIA receptors, OT can target every actor of the thermoregulatory system at both peripheral and central levels. These sites of action are summarized in Figure 1.

## 3. Part 2: Importance of the oxytocinergic system in the sensory processing in the case of ASD, a focus on atypical thermosensory response

### 3.1. The interconnection between the oxytocinergic system and ASD

ASD is characterized by two types of manifestations that could be combined: deficits in communication and social interaction as well as stereotyped behaviors and restricted interests (Diagnostic Statistical Manual of Mental Disorders: *D. S. M*., 2013). Many publications reported a potential dysfunction of the oxytocinergic system as a pathophysiological mechanism of ASD (Insel et al., 1999; Hammock and Young, 2006; Green and Hollander, 2010; Meyer-Lindenberg et al., 2011; Zink and Meyer-Lindenberg, 2012; Lukas and Neumann, 2013; Preti et al., 2014; Lee et al., 2015; Romano et al., 2016) and particularly the importance of the oxytocinergic system during the critical window of the early life regarding the development of ASD (Muscatelli et al., 2018). In addition, dysregulation of the oxytocinergic system has been demonstrated for different genetic mouse models of ASD (see Wagner and Harony-Nicolas, 2018 as a review) such as Fmr1-KO (Francis et al., 2014), Oprm1-KO (Gigliucci et al., 2014), Cntnap2-KO (Peñagarikano et al., 2015), Nlgn-3-KO (Hörnberg et al., 2020), Shank3-KO (Harony-Nicolas et al., 2017; Rajamani et al., 2018), and Magel2-KO (Schaller et al., 2010; Meziane et al., 2015; Fountain and Schaaf, 2016) and also the autism Valproic Acid-induced mouse models (Dai et al., 2018). Moreover, mice harboring alteration of the oxytocinergic system displayed an ASD phenotype (Zhang et al., 2017) that could be partly improved by OT administration. This is the case for the OTR-KO (Ferguson et al., 2000), the OT-KO (Sala et al., 2011), the CD38-KO (a protein of OT release regulation) (Jin et al., 2007), and the Magel2-KO mice (Meziane et al., 2015). In humans, a meta-analysis of 31 studies reported that, children with autism, but not adults, present a lower OT level that could be reliable for some of the social and cognitive deficits (John and Jaeggi, 2021). Many preclinical and clinical studies gave rise to clinical trials to assess whether acute IN administration of OT has beneficial effects on patients with autism. A systematic analysis of 28 studies on the effects of OT IN administration in the treatment of ASD showed beneficial effects on social functioning but nothing relevant for the resting part of the ASD symptoms (Huang et al., 2021). In the case of repeated IN OT administration on patients with ASD, the treatment did not show a really strong effect on improving ASD symptoms (Martins et al., 2022).

Another meta-analysis in which authors analyzed 12 fMRI studies to search for the neural effect of IN OT administration in ASD patients concluded that the treatment can modulate the activation of different brain regions depending on the type of paradigm stimulus but specified that the link between alleviation of the social deficits and the OT-induced activation of this brain network remains unclear (Fathabadipour et al., 2022).

While the results of OT treatment in the case of ASD are not very conclusive on its curative effect, it appears that OT may be beneficial for some ASD symptoms and could be considered a promising treatment. It should be noted that the beneficial effect of this kind of treatment seems to be more pronounced for newborns than for adults (Althammer et al., 2022). Thus, further studies are still needed to assess the value of using OT as a new therapy to treat social impairments in ASD.

### 3.2. Atypical sensory processing in ASD

Patients with ASD present atypical sensory processing with hyposensitivity or hypersensitivity to different sensory modalities which are considered by the diagnostic and statistical manual of mental disorder 5th edition (DSM-5) as an ASD diagnosis criterion (Diagnostic Statistical Manual of Mental Disorders: *D. S. M*., 2013). These sensory perceptions of atypia could be partly explained by morphological changes in the brain, particularly in the thickness of the cortex (Habata et al., 2021). Moreover, sensory atypia occurs in 90% of patients with ASD (Robertson and Baron-Cohen, 2017), affects every sensory system (Marco et al., 2011; Baum et al., 2015; Balasco et al., 2020), is detected before the diagnosis is made (Baranek et al., 2013; Estes et al., 2015; Grzadzinski et al., 2020), is a predicting factor for the lack of high-order socio-cognitive behavior (Hong et al., 2019; Vlaeminck et al., 2020; Park et al., 2021), and is linked with the severity of the ASD phenotype (Baum et al., 2015).

For example, patients with ASD are unable to perceive their environment comprehensively and are more focused on the details of what they see, which deprives them of spontaneously forming an overall image from the elements which constitute it but also gives them a better visual acuity for visuospatial detection task (Shah and Frith, 1983; Plaisted et al., 1998; O'Riordan et al., 2001; Baldassi et al., 2009; Joseph et al., 2009; Kéïta et al., 2010). Related to this capacity that patients with ASD have to focus on the details, data from a meta-analysis of 26 studies highlighted the fact that patients with ASD have an increased activity in temporal and occipital regions important for perception and recognition of objects and a decreased activity in the prefrontal region involved in cognitive functions such as decision making, planification, and execution (Samson et al., 2012). Another characteristic of patients with ASD is their difficulty in eye contact (Senju and Johnson, 2009; Madipakkam et al., 2017), which is essential to emotion perception and the development of sociability (Tong et al., 2021). Some studies showed that hesitancies in eye contact can be improved with IN OT administration in adults with ASD (Guastella et al., 2008; Auyeung et al., 2015; Hubble et al., 2017) which then enhances both their ability to process faces as well as their social interaction and empathy ability (Domes et al., 2013; Kanat et al., 2017). This deficit could come from an unpleasant or even painful excessive arousal resulting from the overactivation of the subcortical system including regions of the superior colliculus, the pulvinar of the thalamus, and the amygdala (Hadjikhani et al., 2017).

The auditive perception is also altered in children with ASD who have difficulty discerning the relative order of two nearby tones and show a delay in the neural responses evoked by these auditory stimuli compared to healthy children. This prolonged latency in auditory responses is considered a criterion of autistic severity (Kwakye et al., 2011). In addition, adult patients do not perceive vocal information in the same way as healthy adults and show difficulties in processing the human voice with other sounds or noises (Gervais et al., 2004).

In the case of tactile perception, it has been shown that patients with ASD present a higher detection threshold for static stimuli (acute stimuli) and a lack of sensitivity to dynamic (which increases in amplitude over time to a detectable threshold) stimuli suggesting aberrant habituation, whereas healthy individuals show poorer detection of dynamic stimuli compared to static stimuli (Puts et al., 2014; Tavassoli et al., 2016). Additionally, the temporal discrimination of successive tactile stimulations seems also to be altered with an increased threshold of detection (Buyuktaskin et al., 2021). Moreover, patients with ASD have an aversion to touch, suggesting that a tactile stimulus, albeit weak, is a source of discomfort indicating a potential hypersensitivity to this type of stimulus (Moore, 2015; Kaiser et al., 2016). This tactile atypia could be explained by an excitation/inhibition imbalance at the level of somatosensory neurons of the DRG (Lipina and Blundell, 2022), modification of somatosensation and somatotopic map (Espenhahn et al., 2021), or even direct alteration of the somatosensory circuit in patients with ASD (Orefice, 2020).

The olfactory system is also impaired in patients with ASD, with deficits in odor identification, odor sensitivity, and odor preference that vary in degree depending on the complexity of the disorder (Crow et al., 2020; Lyons-Warren et al., 2021; Yang et al., 2022). However, the detection seems to not be altered (Lyons-Warren et al., 2021).

Similarly, the taste perception is also atypical in patients with ASD with increased sensitivity to food texture as well as specific taste preferences for acidic tastes and greater sensitivity to aftertastes, leading to selective eating behaviors (Chen et al., 2022; Nimbley et al., 2022).

Furthermore, the integration of multisensory stimuli is atypical in patients with ASD with altered temporal processing of audiovisual multisensory information (Kawakami et al., 2020) that could be related to the atypical neural network (Matsuzaki et al., 2022). It is also the case for visuo-tactile information for which patients with ASD have a smaller peripersonal space that could be linked with some of the social impairment in ASD (Noel et al., 2020).

This sensory atypia is also found in ASD genetic mouse models. For example, mouse models with mutations in Mecp2, Gabrb3, Fmr1, or Shank3 exhibit tactile hypersensitivity (DeLorey et al., 2011; Orefice et al., 2016; Orefice, 2020), Grin2b–/– mice have a hypo reactivity to oral tactile stimulation, leading to reduced suckling response, causing the premature death of these mice (Kutsuwada et al., 1996). Syngap1+/– mice sensory cortex activation induced by whisker stimulation is reduced, showing an alteration of tactile information processing (Michaelson et al., 2018). Shank2–/– mice have hypo reactivity to tactile stimulation as well as reduced nociception to chronic pain (Ko et al., 2016), and Cntnap2–/– mice exhibit an increased pain sensitivity to tactile, thermic, and chemical stimuli (Dawes et al., 2018). The Mecp2 KO, a mouse model of Rett syndrome, displays a decreased amplitude in visually evoked responses in the visual cortex and a decrease in visual acuity (LeBlanc et al., 2015; Banerjee et al., 2016). The Grin1+/– model presents an impaired visual depth perception (Lipina et al., 2022). Electrophysiological recordings of the auditory cortex of Fmr1KO mice revealed an impaired response to auditory tones suggesting hypersensitivity of auditory neurons, a phenotype also present in patients with X-Fragile (Rotschafer and Razak, 2013). Tbr1+/– mice model of ASD displayed altered olfactory discrimination and ASD-related behavior (Huang et al., 2019). In genetic models of ASD, such as FMRP, MeCP2, CAPS2, uPAR, NL3, NPN2, and En-2 KO mice, the GABAergic signaling is impaired with excitation/inhibition balance disturbance, reducing the potential of the animals to make experience-dependent refinement of their sensory circuit (Gogolla et al., 2009).

Thus, patients with ASD are a heterogenous population in which a variety of atypical sensory processing occurs and that could be a determinant factor for the development of the disorder. As we reviewed here, OT is an essential component of the sensory system modulation and also a neuropeptide involved in many aspects of autism, showing thus the interplay between these three components.

### 3.3. The special case of the thermosensory system in ASD

We previously reported that OT has been used as a therapeutical treatment for the socio-cognitive part of the deficits present in ASD. Moreover, some studies tend to show that the sensory deficits in autism could also be rescued by OT treatment, notably for the visual system and the ability to maintain eye contact. Considering both the interaction between OT and the sensory system and the alteration of the oxytocinergic system in ASD, it seems logical that OT treatment could be beneficial to treat sensory atypia in ASD. However, to the best of our knowledge in the literature, not all sensory modalities have been explored to demonstrate a potential overall benefit of OT treatment in autism-related sensory atypia. Thus, in this last part of the review, we focus on the thermosensory system, a sensory system that has received less attention compared to others but is atypical in ASD and is known to be regulated by OT.

Initial studies reported hypersensitivity to thermal pain but normal detection of non-nociceptive temperatures in adults with ASD, whereas the opposite was found in adolescents with a hyposensitivity to non-painful temperatures and pain thresholds to normal temperatures (Cascio et al., 2008; Duerden et al., 2015). However, two recent studies showed that the response to temperature in individuals with autism is atypical with paradoxical heat sensations when a cold stimulus is perceived as hot or burning and an insensitivity to the outside temperature (Duerden et al., 2015; Fründt et al., 2017). In the case of Prader-Willi Syndrome (PWS), patients present a decreased detection threshold for cold temperatures and an increased detection threshold for warm temperatures. In addition, the sensations of pain related to cold and hot temperatures were altered (Priano et al., 2009). This abnormal sensory perception could be due to an impairment of small-fiber sensory nerves in patients with ASD (Chien et al., 2020).

Furthermore, impaired thermoregulation is a widely recognized symptom in PWS (Cassidy et al., 2012) and has been suggested to be linked with fewer OT PVN neurons (Swaab, 1997). Indeed, in PWS, there are many cases of children with high fevers and no infectious causes that in some cases lead to hyperpyrexia that can be fatal (Ince et al., 2005). Severe cases of sudden hypothermia also exist (Watanabe et al., 2003). This phenotype is observed from the first months of life and seems to persist since cases in adolescents have also been described (McVea et al., 2016). Additionally, in Schaaf-Yang Syndrome (SSY), 67% of patients also experience these temperature instabilities that may manifest as excessive sensitivity to cold or hypersudation (McCarthy et al., 2018).

Recently, using an autistic mouse model of SSY (Magel2 mouse), our team showed hyporesponsiveness to cold temperatures with cold-induced USV delay that could be partially restored with acute IN administration of an OT agonist (Da Prato et al., 2022).

Thus, the thermosensory system is another example of a sensory modality in which the perception is atypical for ASD patients and for which evidence seems to show that OT could be a promising therapeutical approach.

## 4. Conclusion

The action of OT/OTR signaling on sensory processing and more particularly on the sensitivity to external temperature in the healthy and ASD case has been reviewed.

This nonapeptide is strongly associated with the development of some ASD symptoms such as sensory atypia. It is an important modulator of all the parameters of the autonomic thermoregulation with action at every level of the circuit including the thermo-effectors. This modulation by OT also concerns the different components of behavioral thermoregulation. Animals receiving external OT administration undergo an upregulation of their thermoregulatory capacity, and animals presenting an alteration of the oxytocinergic system showed deficits of thermoregulation and then difficulties to keep a stable core body temperature. These findings have been extended to behavioral thermoregulation and have been mainly conducted on pups.

While thermosensation is under intensive investigation, there are remaining outstanding issues to be addressed. First, as the LH has been recently brought to light as a major structure for this behavioral thermoregulation, no study has yet investigated a direct effect of the OT on this structure in the case of behavioral thermoregulation. It would then be necessary to find out if the same perennialization of these behaviors is induced by an action of the OT on the LH.

Second, it should be noted that a gap exists between information provided by adult animals compared to pups. Recording physiological parameters are indeed more challenging in pups than in adults because miniaturized tools are not always available. This includes monitoring pups' temperature. In behavioral experiments, the monitoring of body temperature is important because it is a readout of the thermoregulatory response. Rectal probing is not adapted and other methods can be used to monitor temperatures. The implantation of temperature transponders under the skin or even in the peritoneal cavity is a good solution for chronic and accurate measurements. However, this kind of system is mostly applicable to adult mice. Other less invasive techniques such as the use of thermic cameras or infrared thermometers are recommended for both adults and pups and have the advantage to monitor local body change. Another point that has to be raised concerning the thermosensory system is the small number of studies that explore thermal perception and the fact that no study exists in the case of newborns. It would be interesting to see if the thermosensation is a cortical modality and if so which part of the cortex is concerned notably in the case of newborns. Then, it would be possible to confront data from newborns regarding thermosensation with the few published data in adults.

A third question that is still not addressed is the effect of OT on thermosensation. In the same manner, thermosensation is poorly explored in the autistic model. Knowing that deficits of thermic perception have been observed in patients with ASD and mainly during adolescence and that low levels of OT are present in children and adolescents with ASD, it seems appropriate to think that thermic perception deficits could be found in animal models. This could help to better understand the origin of these thermosensory deficits in patients with ASD. Concerning these deficits, many clinical trials using IN OT administration to treat atypical sensory perception in ASD present encouraging results. Therefore, such treatment could be tested for improvement of atypical thermic perception in patients with ASD.

## Author contributions

UZ and VM defined the detailed plan of the review. UZ drafted the initial version. VM revised the article. FM and LC critically read the article. All authors contributed to the article and approved the submitted version.
